# Thoracic endovascular aortic repair for descending thoracic aortic thrombus of a patient with Tolosa-Hunt syndrome

**DOI:** 10.1093/jscr/rjaf208

**Published:** 2025-04-15

**Authors:** Kazuki Morooka, Shuhei Kawamoto, Motoharu Shimozawa, Retsu Tateishi, Fumiya Haba, Shunya Ono, Kosaku Nishigawa, Takeyuki Kanemura

**Affiliations:** Department of Cardiovascular Surgery, IMS Katsushika Heart Center, 3-30-1 Horikiri, Katsushika, Tokyo 124-0006, Japan; Department of Cardiovascular Surgery, IMS Katsushika Heart Center, 3-30-1 Horikiri, Katsushika, Tokyo 124-0006, Japan; Department of Cardiovascular Surgery, IMS Katsushika Heart Center, 3-30-1 Horikiri, Katsushika, Tokyo 124-0006, Japan; Department of Cardiovascular Surgery, IMS Katsushika Heart Center, 3-30-1 Horikiri, Katsushika, Tokyo 124-0006, Japan; Department of Cardiovascular Surgery, IMS Katsushika Heart Center, 3-30-1 Horikiri, Katsushika, Tokyo 124-0006, Japan; Department of Cardiovascular Surgery, IMS Katsushika Heart Center, 3-30-1 Horikiri, Katsushika, Tokyo 124-0006, Japan; Department of Cardiovascular Surgery, IMS Katsushika Heart Center, 3-30-1 Horikiri, Katsushika, Tokyo 124-0006, Japan; Department of Cardiovascular Surgery, IMS Katsushika Heart Center, 3-30-1 Horikiri, Katsushika, Tokyo 124-0006, Japan

**Keywords:** descending thoracic aortic thrombus, acute limb ischemia, thoracic endovascular aortic repair, embolism, Tolosa-Hunt syndrome

## Abstract

Descending thoracic aortic thrombus is a rare condition, often identified during the evaluation for an embolic source. The standard treatment is thoracic aortic replacement; however, its high invasiveness limits patient eligibility. Here, we present the case of a 78-year-old male who presented with acute limb ischemia. Contrast-enhanced computed tomography imaging revealed a floating thrombus in the descending aorta. In addition to thrombectomy for limb salvage, treatment targeting the embolic source was necessary. Thoracic endovascular aortic repair was successfully deployed to address the thrombus source. The patient has remained free of recurrent embolic events and has shown stable progress post-procedure.

## Introduction

Descending thoracic aortic thrombus (DTAT), though rare, is increasingly recognized during the diagnostic evaluation of embolic events [1]. The standard treatment for DTAT is descending thoracic aortic replacement surgery [2]. However, the high invasiveness of this procedure limits its use, particularly in high-risk patients. We present a case involving a highly mobile floating thrombus in the descending thoracic aorta, incidentally identified on contrast-enhanced computed tomography (CT) during the evaluation of acute limb ischemia (ALI). Prompt thrombus retrieval was required to salvage the ischemic limb, while simultaneous management of the embolic source was critical to prevent recurrence. Given the patient’s condition and the advantages of minimally invasive techniques, we opted for thoracic endovascular aortic repair (TEVAR) to immobilize the floating thrombus, followed by thrombus retrieval. This combined approach successfully preserved the affected limb and mitigated the risk of further embolic complications.

## Case report

**Figure 1:**
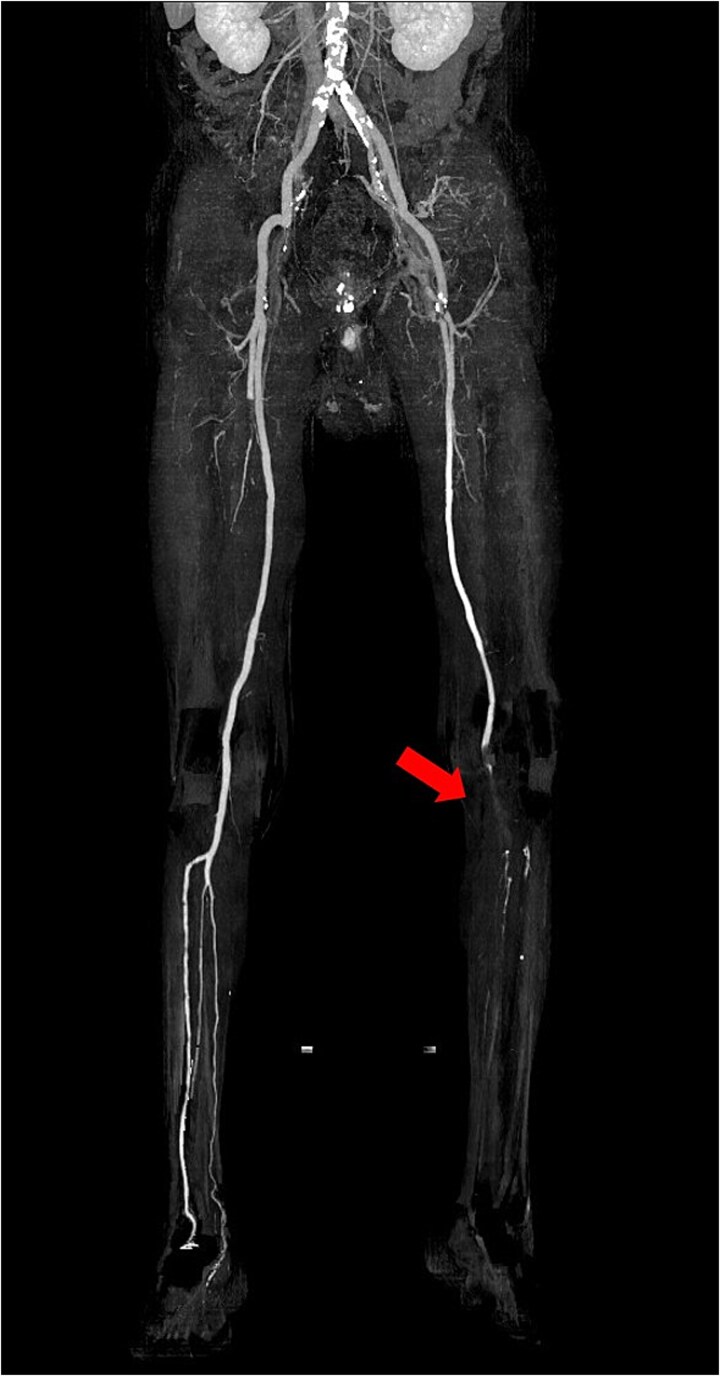
Preoperative 3D reconstructed contrast-enhanced CT. No contrast effect is seen in the blood flow of the lower leg.

**Figure 2:**
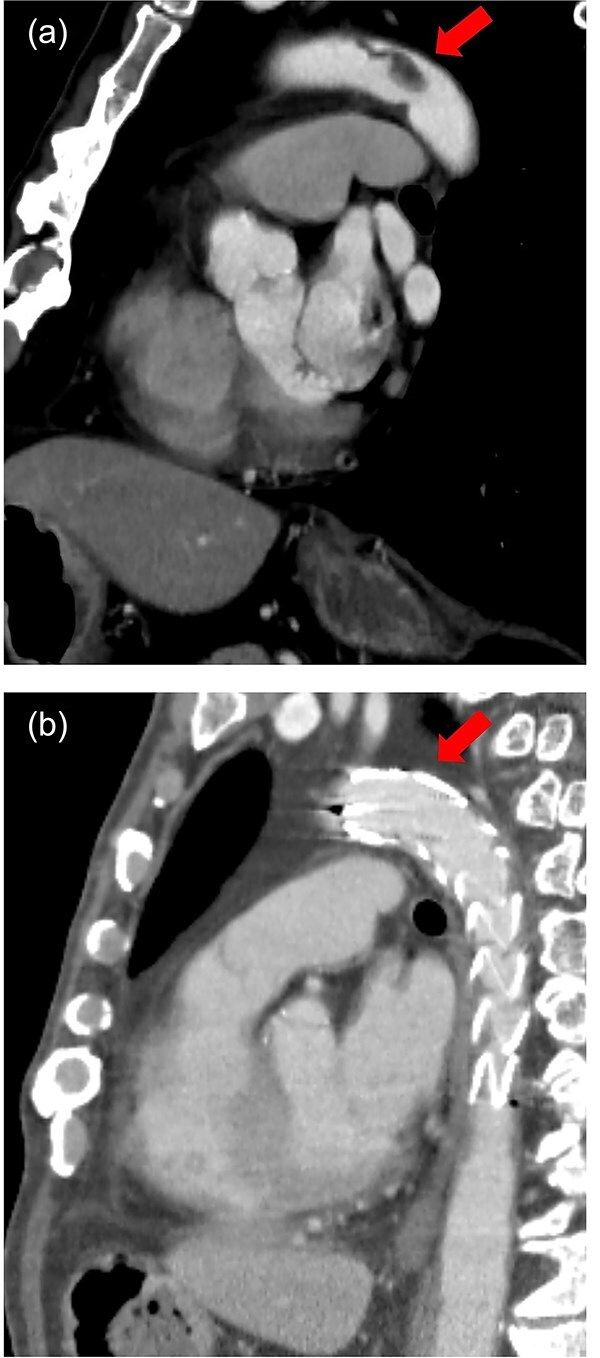
Sagittal view of contrast-enhanced CT. (a) Preoperative. A pedunculated intra-aortic thrombus is attached to the distal arch. (b) Postoperative. Thoracic stent graft is implanted in the distal arch with successful exclusion of the aortic thrombus.

**Figure 3:**
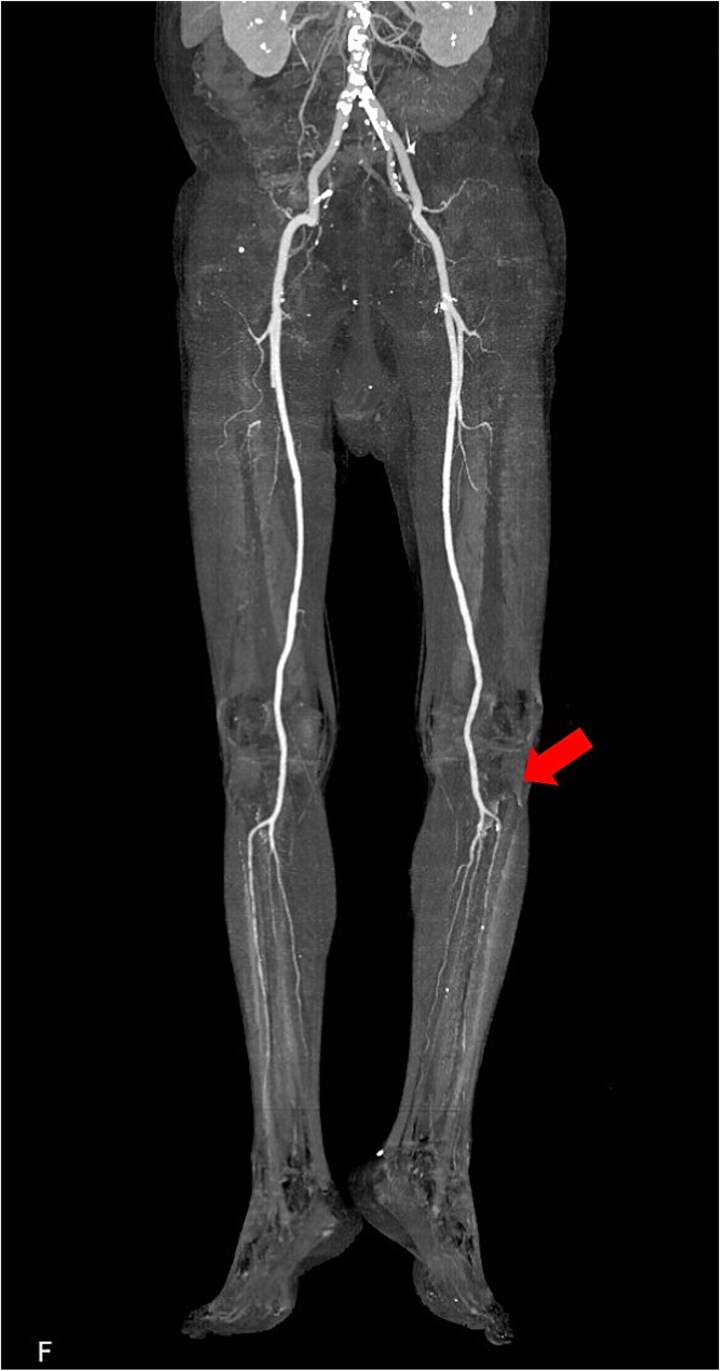
Postoperative 3D reconstructed contrast-enhanced CT. The contrast effect on lower limb blood flow is good.

The patient was a 78-year-old man who had been hospitalized at a prior institution for Tolosa-Hunt syndrome, a rare inflammatory condition causing orbital pain. He had undergone steroid pulse therapy and was maintained on oral prednisolone (40 mg daily). He was also receiving medical treatment for diabetes mellitus, with an HbA1c level of 7.9%. The patient presented with complaints of left leg discomfort and weakness. Contrast-enhanced CT revealed poor perfusion distal to the left common iliac artery, leading to a diagnosis of ALI. He was subsequently transferred to our surgical department for further evaluation and treatment. On arrival, physical examination revealed coldness in the left lower limb, with absent palpable pulses in the dorsalis pedis and posterior tibial arteries. Doppler ultrasonography confirmed the absence of arterial flow in the left lower extremity, consistent with critical ischemia. The patient's electrocardiogram showed sinus rhythm. A review of CT imaging showed thrombotic occlusion extending from the left common femoral artery (CFA) to the superficial femoral artery, with poor perfusion distal to the popliteal artery (Video 1) (Fig. 1). Additionally, a filling defect was identified in the distal aortic arch, consistent with a thrombus (Fig. 2a). The thrombus appeared pedunculated and was attached to the lesser curvature of the descending thoracic aorta distal to the left subclavian artery. It was considered highly mobile, posing a significant risk of embolization. No evidence of intracardiac thrombi was observed, and the DTAT was identified as the likely embolic source responsible for the ALI. Given the urgency of salvaging the ischemic limb, thrombus retrieval was prioritized, while simultaneous management of the embolic source was deemed equally critical to prevent recurrence. Open surgery was considered high-risk due to the patient’s age, diabetes, and chronic steroid use. As a less invasive alternative, we opted for TEVAR to immobilize the floating thrombus. During the procedure, bilateral CFAs were surgically exposed to provide access and enable immediate removal of the embolic source if needed. A transesophageal echocardiogram (TEE) revealed a highly mobile intra-aortic thrombus (Video 2). A 26× 26 × 150 mm stent graft (Valiant Captivia Thoracic Stent Graft; Medtronic, Santa Rosa, CA, USA) was deployed just distal to the left subclavian artery under fluoroscopic guidance. Real-time TEE guidance ensured continuous monitoring of the floating thrombus and confirmed no embolization during intravascular manipulation. Following stent graft deployment, thrombus retrieval was performed via the left CFA using a 4-Fr Fogarty catheter (Fogarty Fortis arterial embolectomy catheter; Edwards Lifesciences, Irvine, CA, USA), successfully retrieving fibrin thrombi. Intraoperative angiography demonstrated restored blood flow in the lower extremity, with improved perfusion extending to the foot. Pulses in the dorsalis pedis and posterior tibial arteries were palpably restored bilaterally. The patient was extubated in the operating room and progressed without any findings suggestive of intestinal or lower extremity ischemia. Postoperative CT confirmed successful exclusion of the aortic thrombus (Fig. 2b) and restoration of adequate lower limb perfusion (Fig. 3). The patient was initiated on oral anticoagulation therapy with edoxaban 30 mg to prevent future thrombus formation and experienced an uneventful recovery. This patient has remained free of recurrent embolic events and has shown stable progress post-procedure.

## Discussion

DTAT, while rare, is a significant cause of cerebral and peripheral embolism [1, 2]. It is often detected through contrast-enhanced CT or TEE during the evaluation of symptomatic embolic events. Due to the limited availability of data, no definitive treatment guidelines exist for managing aortic floating thrombi. While conservative management with anticoagulation therapy has been reported to yield successful outcomes [3], this approach is neither universally recommended nor standardized [4]. In the present case, a highly mobile floating thrombus in the descending thoracic aorta was identified during the evaluation of ALI. Urgent surgical intervention was necessary to address the ALI, while the thrombus’s mobility posed a high risk of recurrent embolization, necessitating immediate treatment of the embolic source [2]. Open surgical intervention was considered; however, the patient’s advanced age, long-term steroid use, and poorly controlled diabetes were significant risk factors for perioperative complications. As a treatment for thoracic aortic aneurysms, TEVAR has been reported to contribute to a shorter recovery period and a reduced risk of complications compared to open surgery [5, 6]. TEVAR was therefore chosen as a minimally invasive approach to stabilize the thrombus [7]. It is important to note that the manipulation of guidewires and stent grafts during TEVAR carries an inherent risk of thrombus dislodgement and embolization. To mitigate this risk, both CFAs were surgically exposed to ensure immediate access for thrombus retrieval if necessary. Real-time TEE monitoring was employed throughout the procedure to track the floating thrombus, ensuring its stability during stent graft deployment. TEVAR allowed for rapid exclusion of the embolic source and successful restoration of limb perfusion, ultimately salvaging the affected limb. Early postoperative rehabilitation facilitated the patient's recovery of ambulatory function. This case highlights the efficacy and safety of TEVAR as a minimally invasive approach for managing aortic floating thrombi associated with ALI, while emphasizing the importance of comprehensive procedural planning and intraoperative precautions.

## Supplementary Material

Video_1_rjaf208

Video2_rjaf208
